# Effect of Recent Abortion Legislation on Twitter User Engagement, Sentiment, and Expressions of Trust in Clinicians and Privacy of Health Information: Content Analysis

**DOI:** 10.2196/46655

**Published:** 2023-05-12

**Authors:** Karl Swanson, Akshay Ravi, Sameh Saleh, Benjamin Weia, Elizabeth Pleasants, Simone Arvisais-Anhalt

**Affiliations:** 1 Department of Medicine University of California San Francisco San Franicsco, CA United States; 2 Department of Biomedical and Health Informatics Children's Hospital of Philadelphia Philadelphia, PA United States; 3 Department of Biomedical and Health Informatics University of Pennsylvania Philadelphia, PA United States; 4 School of Public Health University of California Berkeley, CA United States; 5 Department of Laboratory Medicine University of California San Francisco San Francisco, CA United States

**Keywords:** Roe v Wade, Dobbs v Jackson’s Women’s Health Organization, abortion, family planning, sentiment analysis, women’s rights, twitter, trust, sentiment analysis, natural language processing, legislation, social media, reproductive health care, health information, users

## Abstract

**Background:**

The Supreme Court ruling in *Dobbs v Jackson Women’s Health Organization (Dobbs)* overrules precedents established by *Roe v Wade* and *Planned Parenthood v Casey* and allows states to individually regulate access to abortion care services. While many states have passed laws to protect access to abortion services since the ruling, the ruling has also triggered the enforcement of existing laws and the creation of new ones that ban or restrict abortion. In addition to denying patients the full spectrum of reproductive health care, one major concern in the medical community is how the ruling will undermine trust in the patient-clinician relationship by influencing perceptions of the privacy of patient health information.

**Objective:**

This study aimed to study the effect of recent abortion legislation on Twitter user engagement, sentiment, expressions of trust in clinicians, and privacy of health information.

**Methods:**

We scraped tweets containing keywords of interest between January 1, 2020, and October 17, 2022, to capture tweets posted before and after the leak of the Supreme Court decision. We then trained a Latent Dirichlet Allocation model to select tweets pertinent to the topic of interest and performed a sentiment analysis using Robustly Optimized Bidirectional Encoder Representations from Transformers Pre-training Approach model and a causal impact time series analysis to examine engagement and sentiment. In addition, we used a Word2Vec model to study the terms of interest against a latent trust dimension to capture how expressions of trust for our terms of interest changed over time and used term frequency, inverse-document frequency to measure the volume of tweets before and after the decision with respect to the negative and positive sentiments that map to our terms of interest.

**Results:**

Our study revealed (1) a transient increase in the number of daily users by 576.86% (95% CI 545.34%-607.92%; *P*<.001), tweeting about abortion, health care, and privacy of health information postdecision leak; (2) a sustained and statistically significant decrease in the average daily sentiment on these topics by 19.81% (95% CI −22.98% to −16.59%; *P*=.001) postdecision leak; (3) a decrease in the association of the latent dimension of trust across most clinician-related and health information–related terms of interest; (4) an increased frequency of tweets with these clinician-related and health information–related terms and concomitant negative sentiment in the postdecision leak period.

**Conclusions:**

The study suggests that the *Dobbs* ruling has consequences for health systems and reproductive health care that extend beyond denying patients access to the full spectrum of reproductive health services. The finding of a decrease in the expression of trust in clinicians and health information–related terms provides evidence to support advocacy and initiatives that proactively address concerns of trust in health systems and services.

## Introduction

In May 2022, Politico released [1] a leak of the United States Supreme Court’s draft majority opinion of *Dobbs v Jackson Women’s Health Organization (Dobbs)*, which was later confirmed in the final decision on June 24, 2022 [2]. The decision overruled the precedents established by *Roe v Wade* [3] and *Planned Parenthood v Casey* [4], which guaranteed a pregnant person’s (referred to as “woman” from here on) right to abortion. The *Dobbs* decision allows states to individually regulate access to abortion care services. While many states have passed laws to protect abortion since *Dobbs* [5], this decision has also triggered the enforcement of existing laws and the creation of new ones that ban or restrict abortion. To date, 35 states have legally restricted abortion access [6], creating a national landscape of abortion care that is unclear, uneven across states, and constantly changing. These restrictions limit abortion care delivery and are projected to reduce access to abortion care by 40% [7]. Women who are denied access to desired abortion care have reported worse physical health [8], mental health [9], and socioeconomic outcomes [10] compared to women who are able to access abortion care.

In addition to the far-reaching implications of the *Dobbs* ruling on many aspects of peoples’ lives, one major concern in the medical community is how *Dobbs* will undermine trust in the patient-clinician relationship [11], by influencing perceptions of the privacy of patient health information. We refer to trust as the attitudes that patients have toward their relationship with clinicians. Since the *Dobbs* ruling, there has been increased awareness surrounding how reproductive health data in both traditional health care settings and beyond can be used against patients. For example, reports have detailed the known limitations of major federal regulation, such as the Health Insurance Portability and Accountability Act (HIPAA), in protecting patients’ reproductive health data in the context of patient care [12-15] and how the massive exchange of health care data across organizations and state lines could jeopardize patients who receive abortion care [13,16,17]. Additionally, outside traditional health care settings, reports have detailed how mobile apps collecting data about users’ health, sexual behavior, and menstrual cycles have shared user data without the user’s knowledge or consent [18] and how these data could be used to prosecute women who receive abortion care [19].

While there is limited research exploring patients’ historical trust in clinicians performing abortion, it is known that patients’ trust in reproductive health care clinicians and the recommendations these clinicians give are compromised for patients with marginalized identities and those with trauma experiences [20]. Without trust in clinicians, clinics, or health systems to protect reproductive health data, patients may choose not to disclose information about pregnancy-related vital health information such as their last menstrual period, prior abortions, or previous obstetric complications. To date, the impact of the *Dobbs* decision on social trust in health care systems, clinicians, and privacy of health data, particularly surrounding women’s health, has not been well characterized.

Social media, specifically the web-based microblogging platform Twitter, can be one avenue to explore public sentiment and changes in social trust. With over 235 million daily users reported in 2022 [21], Twitter users represent a sample of public discourse on the *Dobbs* decision and social trust in the health care community and privacy of health data. Prior work has demonstrated the use of Twitter as a tool for health research [22] and to understand trends in social concerns in the context of current events and public health, such as the COVID-19 pandemic [22-26], mental health [27,28], and Affordable Care Act marketplace enrollment [29]. Researchers have also used Twitter to analyze users’ backlash to Georgia’s 2019 abortion ban [30], and the temporal, geographical, and sentiment patterns in the public’s reaction to the overturning of *Roe v Wade* with further stratification between pro-life and pro-choice tweets [31]. Beyond the analysis of unstructured text on social media platforms, advanced text analysis is also becoming increasingly used in the health care domain [32]. In this study, we build upon the existing literature and analyze tweets to better characterize (1) Twitter users’ engagement and sentiment surrounding federal abortion legislation as it relates to abortion, health care, and privacy of health information over time and (2) the *Dobbs* decisions’ impact on the cultural connotations of expressions of trust in clinicians and the privacy of health information.

## Methods

### Ethics Approval

This study was not reviewed by an Institutional Review Board as it was performed on an open and publicly available dataset.

### Overview

**Figure 1:**
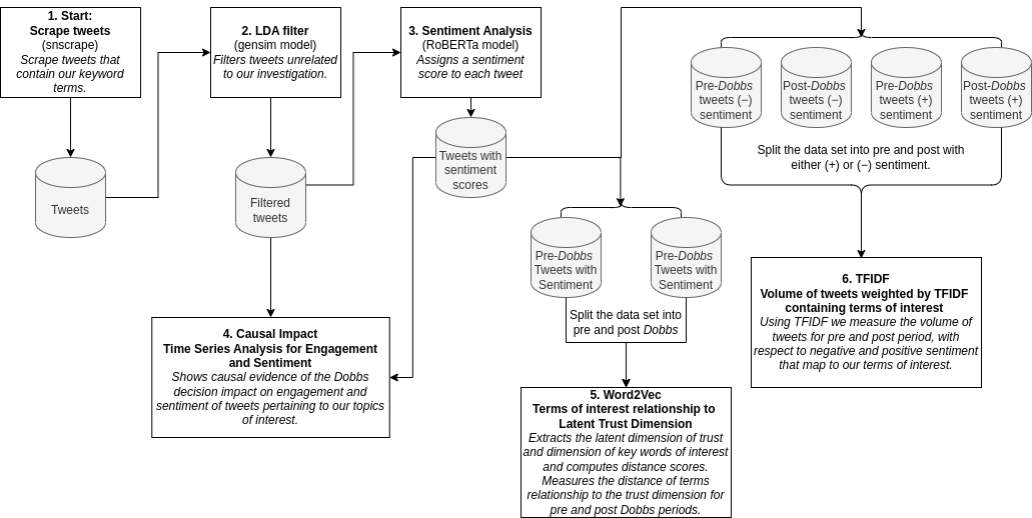
A flowchart illustrating each step in our methods starting from scraping twitter. Cylindrical shapes indicate a dataset and square boxes represent data processing or analysis. LDA: Latent Dirichlet Allocation; RoBERTa: Robustly Optimized Bidirectional Encoder Representations from Transformers Pre-training Approach; TFIDF: term frequency, inverse-document frequency .

We analyzed changes in Twitter engagement and sentiment regarding abortion, health care, and privacy of health information before and after the *Dobbs* decision was leaked to the public. Furthermore, we sought to quantify the cultural connotation of expressions of trust for several reproductive health care and health information–related terms of interest for the predecision leak and postdecision leak periods. The predecision leak period is January 1, 2020, to May 1, 2022, and the postdecision leak period is May 2 to October 17, 2022. These periods correspond to before and after Politico published the draft majority opinion [1]. The postleak period includes the final decision released on June 24, 2022. We will refer to these distinct time periods as preleak and postleak periods henceforth. Figure 1 is a flowchart illustrating each step in our methods (Figure 1).

### Data Collection, Cleaning, and Preprocessing

We used the Python package snscrape (JustAnotherArchivist) [33] to web scrape Twitter for tweets that included the following terms: “Dobbs,” “Jackson,” “Dobbs” & “Jackson,” “Roe,” “Roe” & “Wade,” and the following permutations and combinations of terms (“Dobbs” or “Jackson” or “Roe” or “Wade” or “Abortion”) & (“privacy” or “share” or “HIPAA” or “private” or “disclosure” or “PHI”). The scrape included Tweets posted between January 1, 2020, and October 17, 2022. Duplicate tweets were filtered.

To select the tweets pertaining to the topic of interest, we used Python (version 3.8) and gensim (version 4.3.0) to train a Latent Dirichlet Allocation (LDA) model [34], which clustered our data into 20 topic areas to evaluate for and remove potential confounding topics (eg, Michael Jackson), given that manual filtering was not feasible for the size of our data set. This LDA model was then used to assign probabilities to a given tweet belonging to a certain topic. We selected tweets with a 95% or greater probability of belonging to topics that contained salient terms of interest.

### Determining Preleak and Postleak Twitter Engagement and Sentiment

We computed Twitter engagement and sentiment on the LDA-filtered Tweets in the preleak and postleak periods. Engagement was defined as the average number of unique users per day with tweets including our terms of interest post deduplication and LDA topic filtering. Each tweet’s sentiment was calculated by a Robustly Optimized Bidirectional Encoder Representations from Transformers Pre-training Approach (also known as “RoBERTa”) model, which has been validated in the literature and fine-tuned on Twitter data [35-37]. Sentiment is defined as the model’s highest probability score for each tweet for negative, neutral, and positive sentiment. This model allowed us to encode a sentiment score for each tweet as negative (0), neutral (0.5), and positive (1.0). The encoded sentiment score was then averaged across tweets per day.

We also used the Causal Impact package (version 0.0.13) [38] to fit a Bayesian structural model on the preleak and postleak engagement and sentiment data. This model calculates posterior distributions for both the real-world data and a predicted counterfactual as a quasi-control. We used the posterior distribution to determine if a significant change had occurred in user engagement and sentiment across the preleak and postleak periods compared to the counterfactual quasi-control.

### Cultural Connotations of Expressions of Trust at the Unigram Term Level

We sought to quantify the effect of the *Dobbs* decision on the cultural connotations of expressions of trust, particularly surrounding clinicians and privacy of health information, at the individual word—otherwise known as the unigram term. To do this, we adapted methods from Arseniev-Koehler et al [39]. We first generated word embeddings for these terms by training a Word2Vec [40] skip-gram model on corpora from both preleak and postleak periods. We then extracted a latent semantic dimension of trust from the embeddings. This dimension is the average vector of pairwise synonyms and antonyms of a trust anchor word set. The anchor words for the positive vector space are “trust,” “trustworthy,” “faith,” “reliable,” “confidence,” “certainty,” and “conviction.” The anchor words for the negative vector space are “mistrust,” “untrustworthy,” “distrust,” “unreliable,” “skepticism,” “uncertainty,” and “doubt.” After the dimension was extracted, we projected a target word vector, such as “HIPAA,” onto the dimension and computed its cosine similarity as a scalar, producing a similarity score. The higher the similarity score a term has to the trust dimension, the more likely the term is considered to have a trust connotation and vice versa. The specific unigram level target terms of interest are grouped into clinician terms: “doctor,” “provider,” “physician,” “nurse,” “ob,” “gyn,” “obgyn,” “pcp,” and health information terms: “app,” “information,” “periodtracking,” “tracking,” “PHI” (protected health information), “HIPAA,” “data.” Of note, the term “healthcare” was included in the clinician term set as a measure of trust in health care.

### Term Frequency, Inverse-Document Frequency Volume, and Tweets Containing Terms of Interest for Trust Dimension

We sought to investigate volume changes and the specific example tweets that contain clinician terms and health information terms used in our cultural connotation of expression of trust analysis. To perform this, we first performed the term frequency, inverse-document frequency (TFIDF) [41] on our LDA-filtered data set for tweets that contain our terms. The purpose of the TFIDF is to weight documents by important terms. We were able to leverage TFIDF after we split the data set into tweets with a negative or positive sentiment score and mapped those sets of tweets to each term via TFIDF for the preleak and postleak periods. For visualization purposes, we normalized the volume of tweets on a standard score scale. Finally, we selected tweets that (1) surpassed the TFIDF’s weighted threshold per term and (2) did not contain article headlines and links. We then performed a granular manual review of tweets that matched these criteria and heuristically evaluated if their content applied to the theme of trust in clinician and health care information terms. The volume of tweets identified by the TFIDF for manual review was variable by term, ranging from 6 tweets per term to over 100, depending upon whatever the TFIDF deemed was the most important tweet per term.

## Results

### Data Set and Filtering

**Figure 2:**
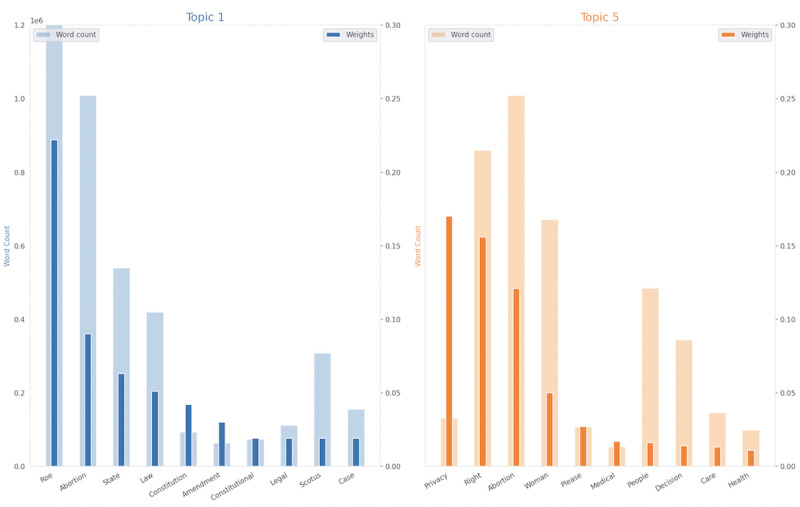
The top 10 weighted terms for Topics 1 and 5 are shown. Tweet membership to these topics served as the inclusion criteria for our final dataset. The weights are specified as a dark bar and word count as a larger light bar.

We collected 14,912,707 tweets with our terms of interest. After removing duplicates, we had 10,360,328 unique tweets. Figure 2 shows the word counts and weights for the most salient terms for LDA topics, labeled “1” and “5” by the LDA model and referred to throughout as “Policy and Legality,” and “Health Privacy,” respectively (Figure 2). Topic 5 had the terms “privacy,” “right,” “abortion,” “woman,” and “please,” as the top 5 most salient terms. Topic 1 had “roe,” “abortion,” “state,” “law,” and “constitution” as its top 5 most salient terms. An interactive version of the topics from our LDA model can be viewed on the internet [42]. After filtering for tweets that only belonged to these topics, our final data set included 1,380,166 tweets.

### Time Series Causal Impact Analysis—Engagement Results

**Figure 3:**
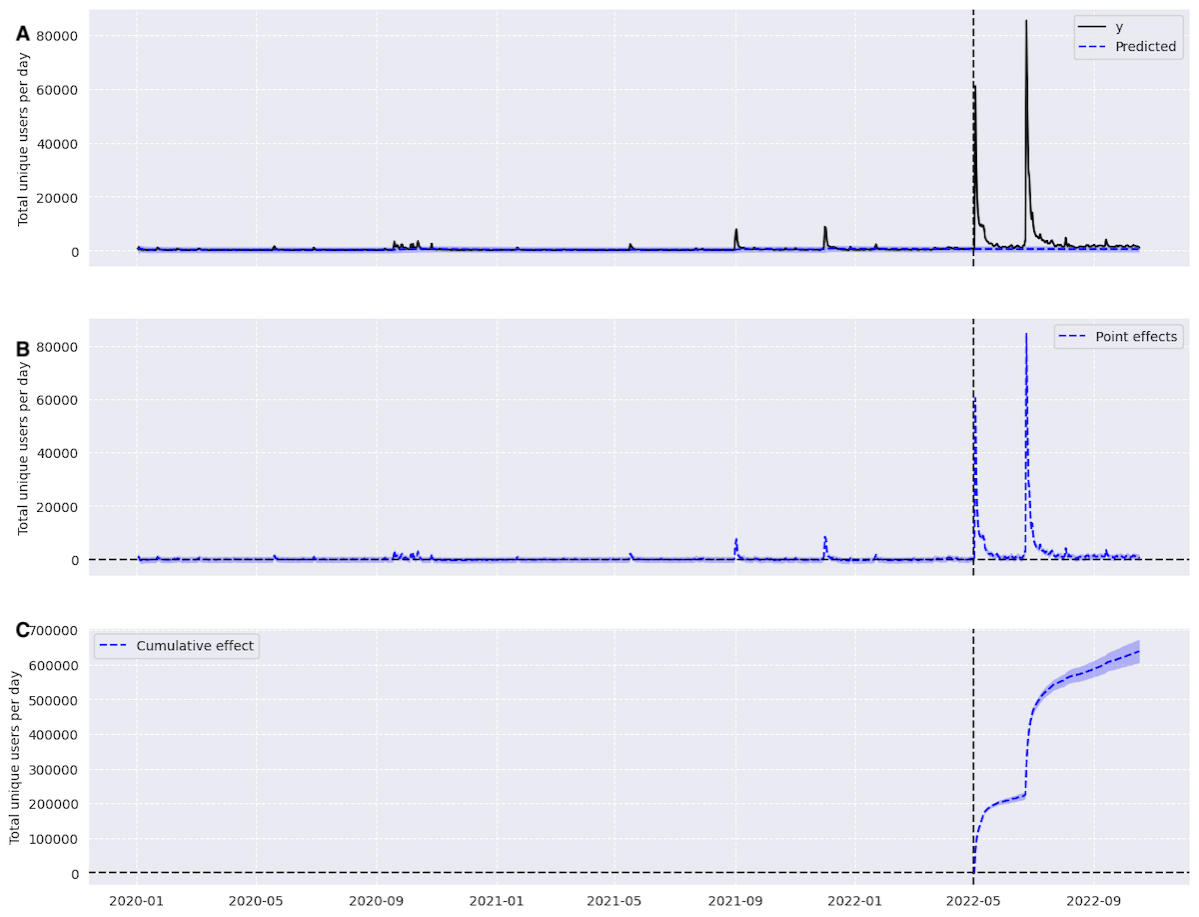
(A) The average number of unique users per day in black and the counterfactual estimate in blue. (B) The point difference in the predicted counterfactual and the actual number of unique users per day, and (C) the cumulative difference in blue, with the CI for all graphs being the blue shadow surrounding the line.

The number of total unique daily users tweeting on topics in our LDA-filtered data set increased in the postleak period (Figure 3). According to the counterfactual estimate based on data from the preleak period, had the *Dobbs* decision not happened, we would have expected 110,552 (95% CI 76,195.37-145,356.23) total unique users and 650.13 (95% CI 448.21-855.04) unique daily users to tweet in the postleak time period. Instead, we observed 748,081 unique users and 4400.48 unique daily users during the postleak time period. The number of unique daily users increased by 576.86% (95% CI 545.34%-607.92%). The probability of obtaining this effect with a Bayesian one-sided tail area probability is *P*<.001. However, this increase was not sustained, as can be seen in (Figure 3A) where within several months post leak, the volume of actual unique daily users matches the predicted volume of daily users.

### Time Series Causal Impact Analysis—Sentiment Results

**Figure 4:**
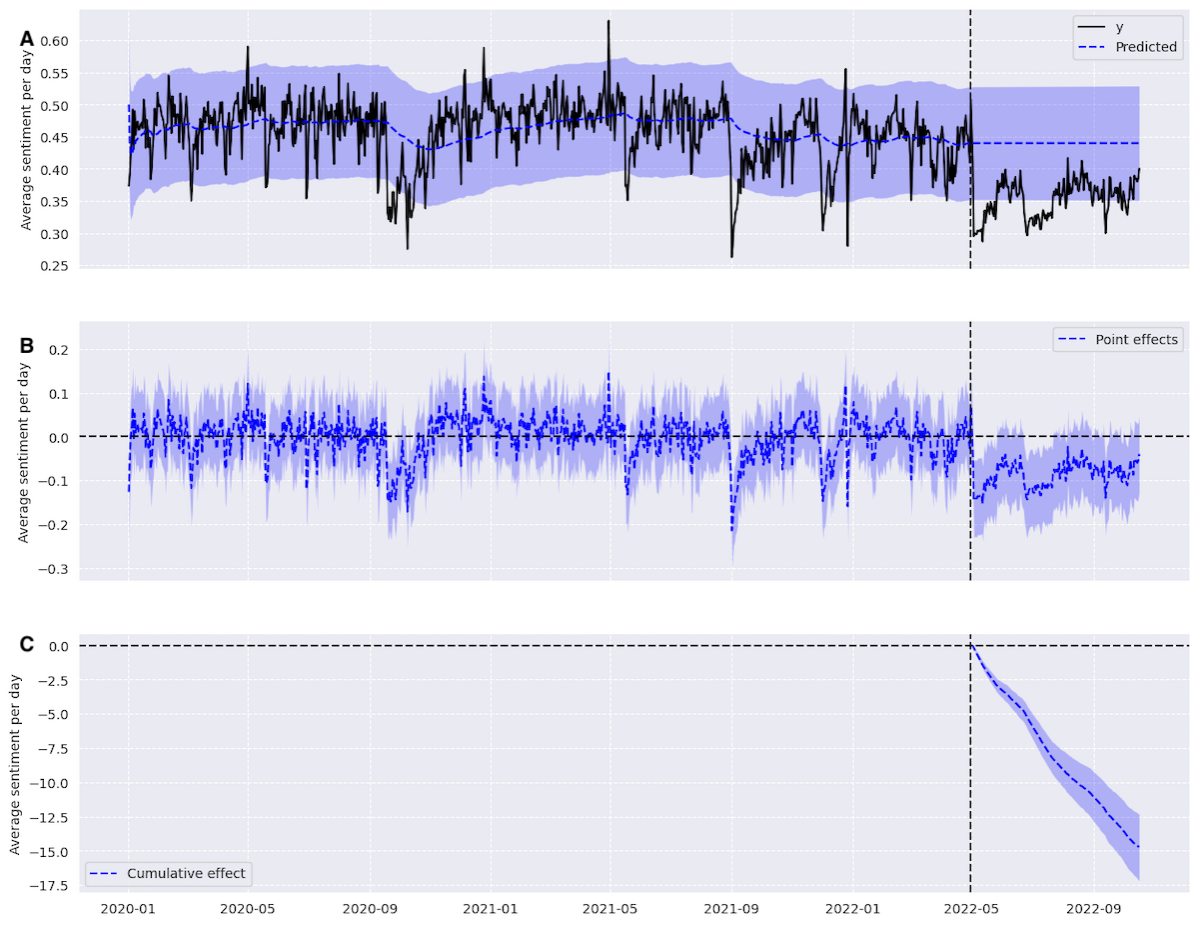
(A) The average sentiment per day in black and the counterfactual estimate in blue, (B) the point difference in the predicted counterfactual and the actual average sentiment per day, and (C) the cumulative difference in blue, with the CI for all graphs being the blue shadow surrounding the line.

The average daily sentiment for tweets on topics in our LDA-filtered data set decreased and became more negative in the postleak period and was persistently negative for several months post leak, as seen in Figure 4. From our counterfactual estimate from the preleak period, we would have expected an average daily sentiment of 0.44 (95% CI 0.43-0.45) or near neutral, had the decision not happened. The observed average daily sentiment was 0.35, a decrease of 19.81% (95% CI −22.98% to −16.59%). The probability of obtaining this effect by chance is minuscule with a Bayesian one-sided tail-area probability (*P*<.001).

### Word2Vec Analysis—Cultural Connotations Results

**Figure 5:**
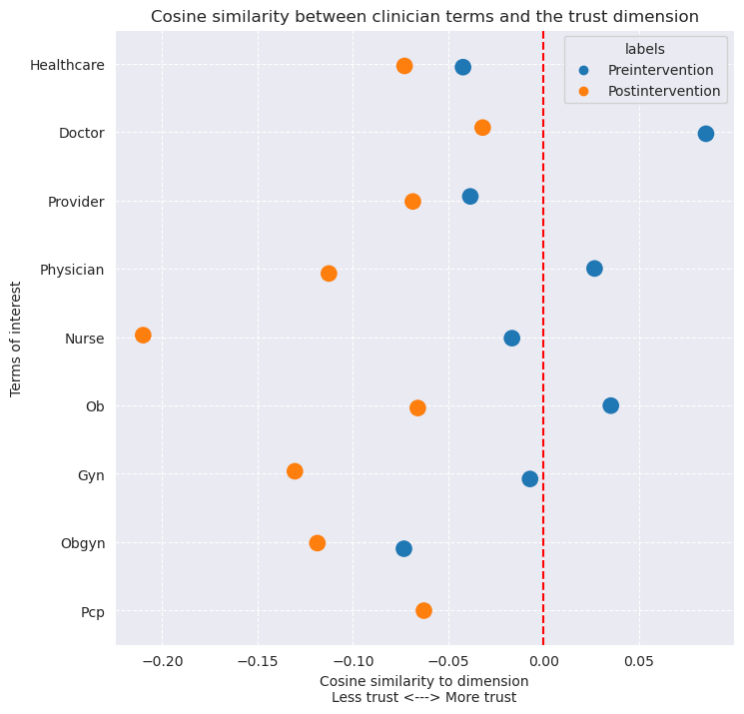
Plot of the terms of interest for the clinician related groups of terms projected onto the trust dimension of our Word2vec models. Blue is "pre" period and orange is the "post" period.

**Figure 6:**
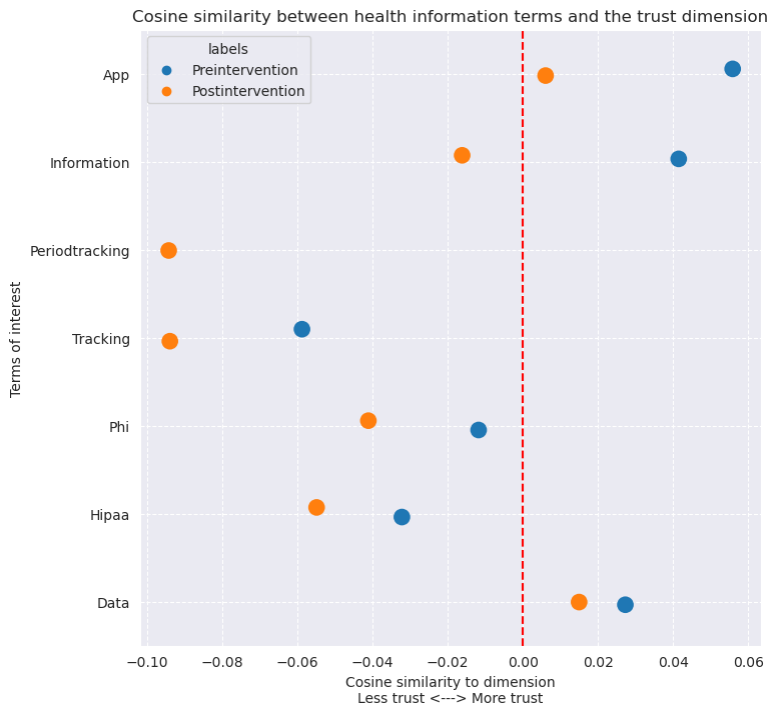
Plot of the terms of interest for the health information related groups of terms projected onto the trust dimension of our Word2vec models. Blue is the "pre" period and orange is the "post" period.

Groups of clinician-related and health information–related terms all show a net decrease in cosine similarity to the trust dimension (Figures 5 and 6). Many terms in the clinician group were already negatively associated with the trust dimension in the preleak period, such as “healthcare,” “provider,” “nurse,” “gyn,” “obgyn,” but these still had an overall reduction in association to the trust dimension in the postleak period. The terms “doctor,” “physician,” and “ob” from the clinician group were initially positively associated with the trust dimension in the preleak period; however, in the postleak period, all terms became negatively associated. Similarly, in the health information term group “tracking,” “PHI,” and “HIPAA” were also negatively associated with the trust dimension in the preleak period and became further negative in the postleak periods. The terms “app” and “information” were positively associated with the trust dimension in the preleak period; however, in the postleak period, these terms became negatively associated. The term “data” remained positive overall but showed a reduction in the postleak period as well. The terms “pcp” from the clinician terms and “periodtracking” from the health information terms did not appear in the preleak period data sets at all; however, in the postleak period, they both had negative associations with the trust dimension.

### TFIDF Results

**Figure 7:**
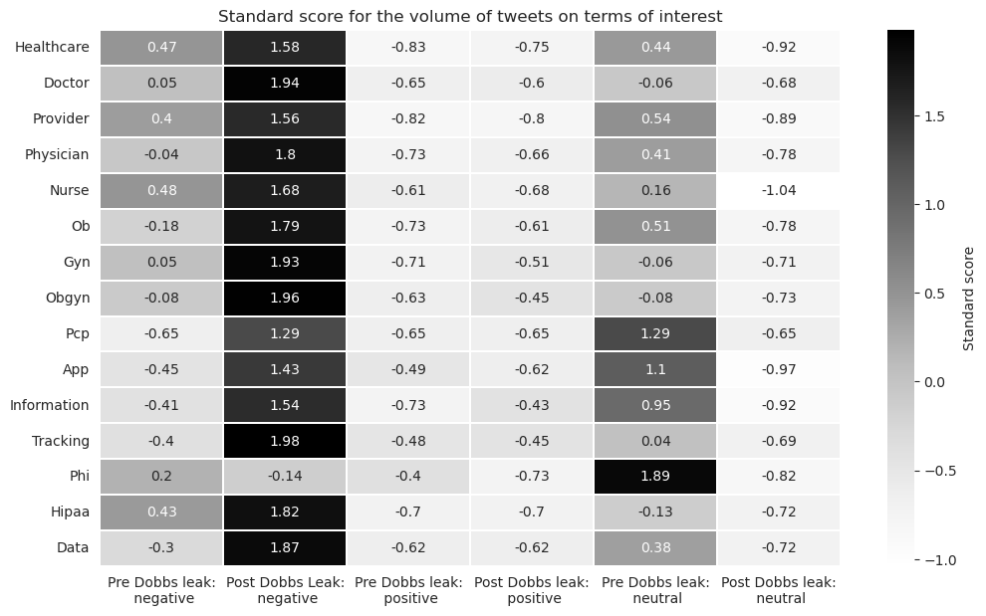
The volume of tweets for a given term of interest for the pre and post periods, split by negative and positive sentiment. In the negative sentiment dataset there is a trend of increased utilization of terms of interest during the post Dobbs period. Where as this phenomena is not observed in the positive sentiment dataset. The volumes were normalized on a standard scale for visualization purposes.

In the postleak period, there is a net increase in tweets that have a negative sentiment and map to our terms of interest, except for the term “PHI” which showed a decrease in volume (Figure 7). This differs from tweets with a positive sentiment, where there is no identifiable pattern for an increase or decrease in our terms of interest. Our investigation of tweets with the highest TFIDF score per term for the negative sentiment postleak period is listed in (Textbox 1).

**Example tweets selected via term frequency, inverse-document frequency for our terms of interest.**

**Healthcare**

- “@user_redacted Overturning Roe gives strangers control of a woman's healthcare.”

**Doctor**

- And this is why we can’t have abortion bans. Because we can’t trust our doctors to not turn us over to the state over private health matters. It’s not just student athletes who get asked about their period during their physicals.

**Provider**

- “This makes no sense. The provider based this choice off the over turning of Roe v Wade, but where is the law that states they can’t prescribe that MEDICATION to anyone? Providers are causing the issue themselves.”

**Physician**

- “@user_redacted @user_redacted @user_redacted @user_redacted @user_redacted You have no idea what what the convos looked like between the physicians and patient, because it’s not your health ans it’s private. The physicians could have said no to protect their job, and that will happen more and more bc of the overturning of Roe, regardless of the laws.”

**Nurse**

- “I have seen too many nurses praise the over turning of roe v wade and that’s???? Super concerning????”

**Ob**

- “@user_redacted Yesterday, a recently retired Ob/Gyn told me that before Roe, the Ob/Gyns were the specialists in sepsis because they saw septic abortions every night they were on call. Guess they should brush up on those skills...”

**Gyn**

- “I don’t know what the future of OB-Gyn looks like without the protections of Roe V Wade. Frankly I’m scared.”

**ObGyn**

- “@user_redacted @user_redacted @user_redacted @user_redacted @user_redacted It was for me until roe was overturned . Now it’s my front burner. Seeing these tweets from OBGYN’s who can’t even perform medical abortions now in some states is just not right. They need lawyers just to proceed with care because the laws are so poorly written. It’s horrific”

**PCP**

- #RoeVWade My wife will not survive a pregnancy. She's on birth control but when 45 was elected I immediately booked a vasectomy because of exactly this.\n\nWhen I got the referral from my pcp she asked why and i said the fascist coup.

**App**

- @user_redacted Roe v Wade overturned in US today making abortion illegal in many states. If you get an abortion for any reason, you could be prosecuted & info in your period tracking app can be used as evidence against you. Contact CS for your app and ask that your data be wiped.

**Information**

- @user_redacted @user_redacted Collateral damage from overturning Roe. When a state can pass laws governing women’s reproductive systems, sharing information on menstrual cycles and birth control usage with a dr could put you in legal jeopardy. Withholding such information from dr could be life threatening.😤'

**Tracking**

- '@user_redacted I hear you. All those states banning abortion, tracking who’s buying contraception, tracking folk going to planned parenthood… yeah them justices giving them judicial approval to do that stuff don’t get to be private any longer”

**PHI**

- “@user_redacted Except that HIPAA makes an exception for disclosure PHI required by other (state) laws. A state could make abortion - even miscarriage - a mandated reporting incident, just like gunshot wounds.”

**HIPAA**

- “@user_redacted @user_redacted @user_redacted menstruation is not protected under HIPAA now that abortion is illegal. you can be investigated for miscarriage, stillbirths, etc which can all be calculated by the dates of a period. knowing if it’s regular is more than enough info”

**Data**

- “Don't get it twisted! Cellphone tracking data will absolutely “be used to target both patients and abortion providers if Roe v. Wade is overturned.” #BansOffOurBodies #AbortionIsHealthcare #AbortionIsARight

## Discussion

### Principal Findings

This study analyzed tweets to characterize Twitter users’ engagement and sentiment in the context of patient trust surrounding the leak of the Supreme Court decision in *Dobbs*. Our study revealed (1) a transient increase in the number of daily users tweeting about abortion, health care, and privacy of health information; (2) a sustained and statistically significant decrease in the average daily sentiment on these topics post leak; (3) a decrease in the association of the latent dimension of trust across most clinician-related and health information–related terms; (4) an increased frequency of tweets with these clinician-related and health information–related terms and concomitant negative sentiment in the postleak period. These study findings are supported by a review of individual tweets from our TFIDF analysis for each term where it is apparent in the postleak period data set that expressions of trust regarding clinicians and the privacy of health information are lacking. Taken together, these results suggest that the *Dobbs* decision’s impact may extend beyond its direct medicolegal implications and could contribute to an insidious erosion of trust in the clinician-patient relationship.

A study by Mane et al [31] similarly assessed the public’s reaction on Twitter following *Dobbs* and found a global increase in conversation about *Roe v Wade* and abortion-related topics. In contrast to our study, Mane et al [31] stratified tweets as “Pro-Life” and “Pro-Choice,” revealing an increase in Pro-Life tweets that persisted longer than Pro-Choice tweets, although there was more negative sentiment overall following the *Dobbs* decision. We replicate and expand on many of Mane et al’s [31] findings, including the increase in Twitter activity discussing abortion surrounding the time of the *Dobbs* decision leak and demonstrate this link through our posterior distribution analysis. Our analysis extends the literature by introducing a novel analysis of cultural connotations of tweets for expressions of trust in various clinician-related and health information–related terms and shows an alarming decrease in trust associated with these terms.

We find that after the *Dobbs* decision, most clinician-related terms suffered a large decrease in association with trust. Notably, the clinician term “nurse” suffered the largest decrease in trust between the preleak and postleak period. This is interesting in the context of how, for the past 20 years, the field of nursing has been considered the profession with the highest rating of honesty and ethical standards in public polling [43]. These changes in perception can have real-world impacts on the delivery of care. Trust in clinicians and health systems, and in the sanctity of the patient-clinician relationship, have been shown to play a role in a patient's access to and use of medical care, adherence to medications and treatment plans, continuity of care, and even self-reported health status [44,45]. Furthermore, similar historical events, like the Tuskegee syphilis study, have shown that these erosions in trust can have long-lasting impacts on patients [46].

We also find a negative impact on the expressions of trust in the privacy of health information. Among clinicians, the medical literature has highlighted the risks associated with how health information, in the traditional care setting or otherwise, can be used to prosecute patients, clinicians, and others who in any form aid in the access to abortion care [13-15,17]. Among the general public, there has been increased attention to and awareness of how apps can collect and misuse data [18,19,47]. Additionally, a recent study of patient perspectives around data privacy revealed that nearly 75% of people are concerned about protecting the privacy of their health data [48]. Our findings support and focus these privacy concerns specifically to the *Dobbs* decision, as patients may be less willing to trust clinicians with reproductive health information and therefore inclined to withhold relevant health information. The clinical implications of missing and incomplete information have been studied in the context of interoperability of health data. Studies have found that the lack of data sharing across health systems and clinical teams hinders and even endangers appropriate clinical care [49-51]. Patients unwilling to share complete and relevant health information due to this erosion of trust may have similar impacts on care.

From a technical perspective, this study demonstrates a different approach to analyzing Twitter activity using natural language processing modeling techniques compared to prior work that queried tweets based on a hashtag for abortion legislation and manually labeled tweet content and themes [30]. We used an unsupervised machine learning technique, LDA, to identify common topics among a cohort of tweets, allowing us to capture tweets that may not have a hashtag associated with them. In fact, in our manual evaluation of tweet contents related to our terms of interest illustrated in Textbox 1, only 1 out of the 15 tweets would have been captured by a hashtag. A similar LDA approach has been applied to COVID-19–related tweets, describing the changes in content and sentiment during the early stages of the pandemic [24]. This study also uses a novel approach to analyzing expressions of trust. This is the first study to our knowledge that studied the cultural connotations of expressions of trust by analyzing the content of tweets at the unigram term level. Moving forward, this analytic technique could be used as a tool to analyze expressions of trust in other forms of writing or speech.

There are several potential limitations to internal validity and generalizability: (1) the list of keywords to start our scrape of Twitter data was selected to be comprehensive, but it still may have missed alternative terminology or misspellings. This may have introduced some selection bias in the tweets related to abortion and privacy that we identified. Additionally, scraping is not meant to be thorough or include a full representative set of tweets; therefore, some relevant tweets may have been excluded from our sampling. (2) There may be topics other than privacy related to abortion and health care that have increased engagement. We mitigate this by filtering our data set via LDA probability to ensure that we are analyzing tweets most related to these topics. (3) The results of the sentiment analysis help show a change in sentiment, as opposed to absolute sentiment, as the absolute sentiment on Twitter is likely to be biased toward a negative sentiment. (4) The measurement of public interest and sentiment toward abortion and privacy based on Twitter is limited by capturing a subset of populations that are present on Twitter. Therefore, it may fail to represent the engagement and sentiments of social groups who are disproportionately not on social media platforms, such as those of lower socioeconomic status with less access to personal devices, or any other population of persons who are otherwise not on social media. (5) In our LDA filtering, tweets that were not written in English were grouped into their own topics, and therefore excluded from our study, so our conclusions may not be generalizable to populations whose preferred language is not English.

### Conclusions

Beyond obstructing access to the full spectrum of reproductive health care and compromising reproductive autonomy, the *Dobbs* ruling has broader consequences for health systems and reproductive health care. This study revealed a statistically significant increase in the number of daily users tweeting about abortion, health care, and privacy of health information and a statistically significant decrease in the average daily sentiment surrounding these topics that were persistent throughout the postleak period. The study also revealed the negative impact of the *Dobbs* decision on the cultural connotation of expressions of trust in clinicians and health information–related terms. The finding of a decrease in the expression of trust in clinicians and health information–related terms provides evidence to support advocacy and initiatives that proactively address concerns of trust in health systems and services.

## Abbreviations

Dobbs: Dobbs v Jackson Women’s Health Organization
HIPAA: Health Insurance Portability and Accountability Act
LDA: Latent Dirichlet Allocation
PHI: Protected Health Information
TFIDF: term frequency, inverse-document frequency
